# N-3 Polyunsaturated Fatty Acids and the Resolution of Neuroinflammation

**DOI:** 10.3389/fphar.2019.01022

**Published:** 2019-09-13

**Authors:** Corinne Joffre, Charlotte Rey, Sophie Layé

**Affiliations:** ^1^INRA, Nutrition et Neurobiologie Intégrée, UMR 1286, Bordeaux, France; ^2^Université de Bordeaux 2, Bordeaux, France; ^3^ITERG, Nutrition Health and Lipid Biochemistry Department, Canéjan, France

**Keywords:** n-3 long-chain PUFAs, docosahexaenoic acid, eicosapentaenoic acid, specialized pro-resolving mediators, nutrition, neuroinflammation, resolvins

## Abstract

In the past few decades, as a result of their anti-inflammatory properties, n-3 long chain polyunsaturated fatty acids (n-3 LC-PUFAs), have gained greater importance in the regulation of inflammation, especially in the central nervous system (in this case known as neuroinflammation). If sustained, neuroinflammation is a common denominator of neurological disorders, including Alzheimer’s disease and major depression, and of aging. Hence, limiting neuroinflammation is a real strategy for neuroinflammatory disease therapy and treatment. Recent data show that n-3 LC-PUFAs exert anti-inflammatory properties in part through the synthesis of specialized pro-resolving mediators (SPMs) such as resolvins, maresins and protectins. These SPMs are crucially involved in the resolution of inflammation. They could be good candidates to resolve brain inflammation and to contribute to neuroprotective functions and could lead to novel therapeutics for brain inflammatory diseases. This review presents an overview 1) of brain n-3 LC-PUFAs as precursors of SPMs with an emphasis on the effect of n-3 PUFAs on neuroinflammation, 2) of the formation and action of SPMs in the brain and their biological roles, and the possible regulation of their synthesis by environmental factors such as inflammation and nutrition and, in particular, PUFA consumption.

## Introduction

Inflammation is a critical process in host defense, facilitating tissue repair, regeneration and maintenance of homeostasis. However, if uncontrolled, it becomes a chronic low-grade inflammation that is characterized by the production of pro-inflammatory cytokines and adipokines leading to tissue damage and loss of function (López-Vicario et al., 2016). This chronic low-grade inflammation is observed in many chronic pathologies including neurodegenerative diseases and also in aging. Hence it becomes a significant public health concern and constitutes a financial burden that impacts millions worldwide (Serhan, 2017b). It is thus of importance to find treatments that ensure resolution of inflammation in a specific time-limited manner. Nutrition has gained importance in recent years since nutriments such as n-3 polyunsaturated fatty acids (PUFAs) have anti-inflammatory properties (Calder, 2016; Calder et al., 2017). They exert their effect in part through their conversion into bioactive lipid mediators called specialized pro-resolving mediators (SPMs) that underlie most of the beneficial effects attributed to their precursors (Serhan, 2014; López-Vicario et al., 2016). Recently, attention has been paid to these derivatives in the regulation of neuroinflammation. In this review, we present an overview of brain n-3 PUFAs and their effect on neuroinflammation and of the formation and mechanisms of the action of SPMs in the brain.

## Brain N-3 PUFAs as Precursors of SPMs

### Brain n-3 PUFAs

The brain contains high levels of PUFAs (25–30%) that are mainly docosahexaenoic acid (DHA, n-3 PUFA) (12–14% of total fatty acids) and arachidonic acid (AA, n-6 PUFA) (8–10% of total fatty acids) (Carrie et al., 2000; Xiao et al., 2005; McNamara and Carlson, 2006; Little et al., 2007; Chung et al., 2008; Joffre et al., 2016). There are regional differences: the hippocampus and prefrontal cortex contain the highest DHA content whereas the hypothalamus has the lowest (Carrie et al., 2000; Xiao et al., 2005; Joffre et al., 2016). There are also cellular differences: astrocytes, oligodendrocytes, and microglial cells (representing respectively 70%, 10–15% and 10–15% of brain glial cells; Renaud et al., 2015) contain DHA in different proportions. DHA represents 10–12% in astrocytes, 5% in the oligodendrocytes and up to 2% in microglial cells (Bourre et al., 1984; Rey et al., 2018). Neurons cannot synthesize long-chain PUFAs (LC-PUFAs) but can incorporate them in their membranes: DHA represents 8.3% of the total fatty acids in neurons (Bourre et al., 1984).

Numerous studies have discussed the transport of DHA through the blood–brain barrier (BBB). DHA enters the brain as unesterified DHA that is the major pool supplying the brain with DHA. However, the precise mechanisms of entry are still not fully described. Some transporters facilitate the uptake of DHA into the brain: fatty acid transport proteins (FATPs), fatty acid translocase (CD36) and major facilitator superfamily domain containing 2A (MFSD2A) (Chouinard-Watkins and Bazinet, 2018). Fernandez et al. (2018) recently reported that a member of the long-chain acyl-CoA synthetase (ACSL) family, ACSL6, is also involved in brain DHA uptake but its role has yet to be determined (Fernandez et al., 2018).

This brain fatty acid composition can be affected by environmental factors such as nutrition, something to which individuals are continuously exposed. Indeed, the PUFA content in all brain structures is strongly impacted by the PUFAs present in the diet (Alashmali et al., 2016; Joffre et al., 2016). A diet rich in n-3 PUFAs (DHA found in fish or its precursor, alpha-linolenic acid, found in vegetable oil) increases brain DHA in rodents (Hiratsuka et al., 2009; de Theije et al., 2015; Skorve et al., 2015; Kitson et al., 2016). However, DHA supplementation is more effective than alpha-linolenic supplementation in increasing the DHA content in the brain (Lacombe et al., 2017; Rey et al., 2019). In rodents, DHA supplementation from 16 weeks to 16 months or from 20 to 22 months of age compensates a DHA decrease due to aging (Little et al., 2007; Labrousse et al., 2012; Bascoul- Colombo et al., 2016). On the other hand, a diet deficient in n-3 PUFAs decreases brain DHA in all brain structures, with the hippocampus, containing most DHA, being the most affected and the hypothalamus the least affected (Delpech et al., 2015b; Joffre et al., 2016; Manduca et al., 2017).

Studies from Broadhurst and Crawford suggest that the amount of DHA incorporated into the brain depends on the complexity of the brain structure and on behavior development (Crawford et al., 1999; Broadhurst et al., 2002). A decrease in brain DHA induced by a deficient diet during gestation and lactation can be reversed by 2-month DHA supplementation at weaning (Orr et al., 2013). The organism also adapts its metabolism to an n-3 PUFA deficiency condition: the half-life of DHA increases in the brain to reduce its loss (Rapoport et al., 2007) and the activity of the enzymes responsible for DHA conversion, the ∆6 desaturase and elongase, is increased in the liver (Cho et al., 1999; Wang et al., 2005; Igarashi et al., 2007). Brain cells are also impacted by dietary PUFA supply. An n-3 PUFA deficient diet decreases DHA in astrocytes, neurons and oligodendrocytes (Bourre et al., 1984) whereas n-3 PUFA supplementation increases DHA levels in glial cells (Bowen and Clandinin, 2005; Destaillats et al., 2010). Moreover, we recently showed that the fatty acid composition of the microglial cells is also modulated by n-3 PUFA dietary intake during the gestation/lactation period. Maternal n-3 LC-PUFA dietary supplementation during gestation and lactation increases the DHA level in the offspring’s microglia at P21 as compared with a maternal diet that contains equilibrated levels of n-6 and n-3 PUFA precursors (Rey et al., 2018). Interestingly, maternal n-3 PUFA deficiency does not impact the DHA level suggesting that microglial cells are protected from n-3 PUFA deficiency (Rey et al., 2018).

### n-3 PUFAs as Regulators of Neuroinflammation

n-3 PUFAs have powerful anti-inflammatory properties (Calder, 2005). They play an important role in the regulation of the synthesis and release of pro-inflammatory mediators (Delpech et al., 2015b; Hanisch and Kettenmann, 2007; Cunningham and Sanderson, 2008; Yirmiya and Goshen, 2011; Pascual et al., 2012). Pro-inflammatory factors include interleukin-1 beta (IL-1β), interleukin-6 (IL-6) and tumor necrosis factor alpha (TNF-α) and play a role in neuronal plasticity (Delpech et al., 2015b; Yirmiya and Goshen, 2011). If sustained, uncontrolled inflammation can lead to neuronal damage that is involved in many neuronal pathologies (Blais and Rivest, 2003; Layé, 2010; Solito and Sastre, 2012). Hence, limiting the inflammation and enhancing the resolution of inflammation is of great interest.

#### Evidence in Humans

In humans, the anti-inflammatory properties of n-3 PUFAs were first identified in epidemiological studies in Eskimos that consume a lot of n-3 LC-PUFAs from eating fish (Dyerberg and Bang, 1979; Kromann and Green, 1980; Simopoulos, 2008). Clinical studies have highlighted the beneficial effect of n-3 LC-PUFAs in chronic inflammatory and autoimmune diseases. Indeed, fish oil supplementation decreases pro-inflammatory cytokine expression, such as IL-1β in blood monocytes, and improves the symptoms of patients suffering from rheumatoid arthritis (Kremer et al., 1990; James et al., 1997; Kremer, 2000) or multiple sclerosis (Stewart and Bowling, 2005; Weinstock-Guttman et al., 2005). Moreover, DHA supply significantly decreases the circulating inflammatory markers and the oxidative stress (Freund-Levi et al., 2006; Kiecolt-Glaser et al., 2012). DHA supply for several months also improves the working and long-term memories in patients with moderate cognitive alterations (Freund-Levi et al., 2006; Lee et al., 2013).

#### Evidence in Animals

In animals, numerous studies have demonstrated the anti-inflammatory properties of n-3 PUFAs in the brain. In lipopolysaccharide (LPS)-, or IL-1β-, induced inflammation models, dietary n-3 LC-PUFA supplementation in adulthood prevents LPS-induced hippocampal increase of pro-inflammatory cytokines IL-1β and TNF-α in rats and mice (Orr et al., 2013; Dehkordi et al., 2015; Rey et al., 2019). A dietary supply in eicosapentaenoic acid (EPA) decreases the production of LPS-induced pro-inflammatory cytokines IL-1β, IL-6 and TNF-α in the hippocampus and increases the production of anti-inflammatory cytokines IL-10 and IL-4 in both rats (Kavanagh et al., 2004; Lonergan et al., 2004; Dong et al., 2017) and mice (Shi et al., 2016). This modification is associated with a decrease in the phosphorylation of c-Jun and c-Jun N-terminal kinase proteins and in nuclear factor-kappa B (NFκB) that regulate inflammation (Lonergan et al., 2004; Shi et al., 2016). EPA dietary supply attenuates the activation of microglial cells and astrocytes triggered by an intracerebral IL-1β administration and increases the production of IL-10 in the hippocampus in rats (Song and Horrobin, 2004; Song et al., 2008; Dong et al., 2017). Following dietary DHA supplementation, there is an increase of DHA in the phospholipid and free fatty acid fractions. However, only the unesterified DHA is necessary to attenuate neuroinflammation. Nevertheless, the DHA phospholipid pool is an important source of unesterified DHA (Orr et al., 2013). On the contrary, in mice, an n-3 PUFA deficiency from the first day of gestation to weaning increases the expression of pro-inflammatory markers in the hippocampus and an alteration in the motility and the phenotype of microglial cells and alters synaptic plasticity (Lafourcade et al., 2011; Madore et al., 2014; Thomazeau et al., 2017). Using the model of intraperitoneal (ip) injection of LPS, we showed that IL-6 expression is strongly induced in n-3 PUFA-deficient mice whereas sickness behavior is down-regulated *via* the impairment of IL-6 signaling in the brain (Mingam et al., 2008). Moreover, when the deficiency continues in adulthood, it alters GABAergic, dopaminergic and cholinergic neurotransmission (Zimmer et al., 2000; Aid et al., 2003; Chalon, 2006; Larrieu et al., 2014) and anxiety-related, depressive-like behaviors that can be compared with autistic behavior in adults (Mingam et al., 2008; Larrieu et al., 2012; Larrieu et al., 2014; Hughes et al., 2016; Manduca et al., 2017). Following ip injection of LPS, n-3 PUFA-deficient mice display altered hippocampal synaptic plasticity that likely contributes to spatial memory impairment and higher glucocorticoid levels (Delpech et al., 2015b). On the contrary, an n-3 PUFA dietary supply improves emotional behavior alteration and memory deficit in rats (Song and Horrobin, 2004; Song et al., 2008; Dong et al., 2017), restores social and memory performance altered in autism models and improves depressive symptoms in both mice (Pietropaolo et al., 2014; Madore et al., 2016; Weiser et al., 2016) and rats (Bove et al., 2018; Morgese et al., 2018). Of note, n-3 LC-PUFA dietary supplementation attenuates depressive symptoms in depressed patients presenting inflammation (McNamara, 2015; Rapaport et al., 2016; Larrieu and Layé, 2018; Layé et al., 2018). This protective effect seems to be linked to EPA which could target microglia to reduce neuroinflammation (Bazinet et al., 2019). n-3 LC-PUFAs could also modulate neuroinflammation through their effect on the HPA axis (Larrieu et al., 2014; Hughes et al., 2016; Larrieu and Layé, 2018). With Alzheimer’s disease, β-amyloid triggers depressive symptoms through neuroinflammatory processes that could be improved by dietary n-3 PUFAs (Bove et al., 2018; Morgese et al., 2018). Altogether these data reinforce the importance of n-3 PUFAs as regulators of inflammation and associated depressive symptoms.

Another way to modulate neuroinflammation is to administer n-3 PUFAs directly into the brain or peripherally. Indeed, a 24-hour intracerebroventriculary (icv) DHA brain infusion attenuates hippocampal neuroinflammation initiated by icv LPS in mice (Orr et al., 2013). In a mouse model of cerebral ischemia, DHA icv administration inhibits NFκB activation and cyclooxygenase-2 (COX-2) expression in the hippocampus (Marcheselli et al., 2003). Moreover, intrathecal injection of DHA decreases microglial activation, mitogen-activated protein kinase (MAPK) phosphorylation and the production of pro-inflammatory cytokines in the spinal cord of mice (Lu et al., 2013). In addition, in a model of traumatic brain injury in rats, n-3 PUFA ip injection attenuates microglial-induced inflammation by inhibiting the NFκB pathway (Chen et al., 2017).

Dietary n-3 LC-PUFA supplementation requires the use of fish oil. However, fish oil may provide confusing factors such as vitamins, for example. Thus, the use of Fat-1 transgenic mice that convert n-6 to n-3 PUFAs through a desaturase from *C. elegans* is a model that enables the elimination of confusing factors provided by the diet (Kang et al., 2004). Such mice have higher DHA levels in the hippocampus and cortex (Delpech et al., 2015a; Boudrault et al., 2010; Orr et al., 2013). They express less COX-2 (involved in the production of lipid mediators) in the cortex than wild type mice (Boudrault et al., 2010). They are protected against cognitive deficits induced by ip LPS injection through a decrease in neuroinflammation (Orr et al., 2013; Delpech et al., 2015a). This is associated with a decrease in microglial activation (Orr et al., 2013).

#### Evidence in *In Vitro* Studies

*In vitro* studies have shown that n-3 PUFAs have an inhibitory effect on the production of pro-inflammatory cytokines in microglial cells. Indeed, in the microglial cell line or primary culture microglial cells, DHA prevents LPS-induced NKκB activation and then cytokine production by inhibiting LPS receptor presentation and decreases the oxidative stress, nitric oxide (NO) production and inducible nitric oxide synthase (iNOS) expression (De Smedt-Peyrusse et al., 2008; Lu et al., 2010; Antonietta Ajmone-Cat et al., 2012; Pettit et al., 2013; Chen et al., 2014; Corsi et al., 2015; Fourrier et al., 2017; Inoue et al., 2017;). Moreover, DHA prevents LPS-induced MAPK phosphorylation (MAPK pathway playing essential roles in the expression of inflammatory molecules) and induces peroxisome proliferator-activated receptor (PPAR)-γ nuclear translocation that exerts anti-inflammatory effects (Antonietta Ajmone-Cat et al., 2012). Of interest, EPA also affects the production of TNF-α, IL-6 and NO by inhibiting NFκB phosphorylation *via* sirtuin-1 (SIRT-1) (Moon et al., 2007; Chen et al., 2014; Inoue et al., 2017). It is likely that DHA attenuates the inflammatory response in LPS-activated microglia by remodeling lipid bodies that are dynamic organelles in which DHA is incorporated and by altering their interplay with mitochondria and other associated organelles (Tremblay et al., 2016). In addition, DHA and EPA are able to enhance myelin or amyloid β (Aβ) peptide phagocytosis that is associated with a shift in microglial polarization toward the beneficial M2 phenotype and to a decrease in pro-inflammatory cytokine production (Hjorth et al., 2013; Chen et al., 2014).

**Figure 1:**
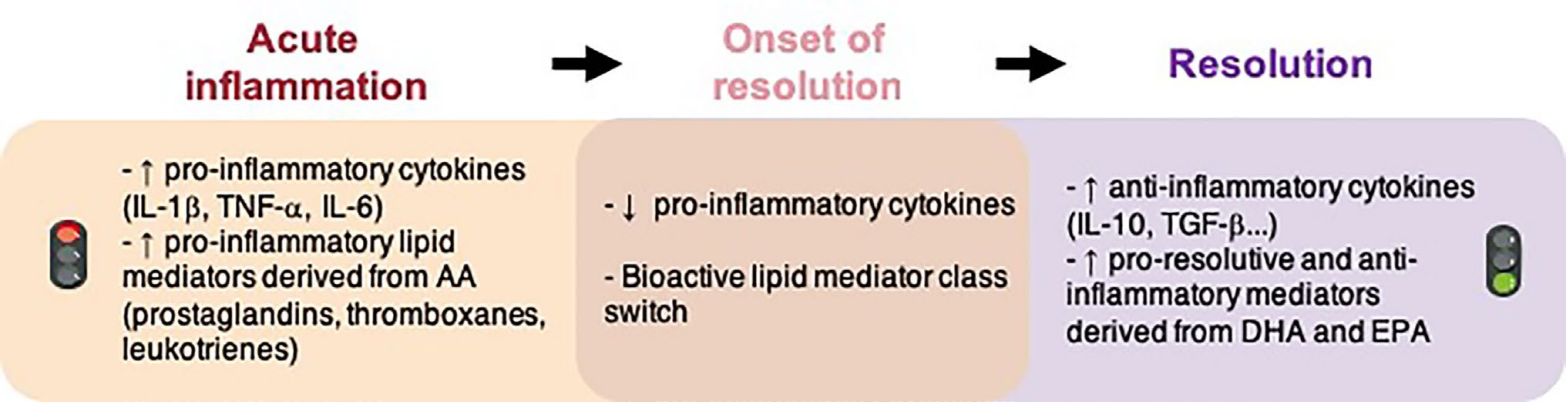
Molecular events implicated in inflammation and the resolution of inflammation. AA, arachidonic acid; DHA, docosahexaenoic acid; EPA, eicosapentaenoic acid; IL, interleukin; TGF, transforming growth factor; TNF, tumor necrosis factor.

This modulation of neuroinflammation induced by n-3 LC-PUFA supply is attributed, in part, to SPM synthesis (Barden et al., 2016). In the various phases of inflammatory response, prostaglandins, leukotrienes and thromboxane are synthesized first (**Figure 1**). They permit the propagation of inflammation. They also stimulate the synthesis of SPMs with pro-resolutive properties (Levy et al., 2001; Serhan and Savill, 2005).

### n-3 PUFA-Derived SPMs and Their Synthesis Pathway

Many of the n-3 PUFA-derived immunomodulators that orchestrate the inflammatory response are lipids (Doyle et al., 2018). Some of them are involved in the regulation of inflammation (prostaglandins, thromboxanes, leukotrienes, etc.) and others are implicated in the resolution of inflammation (resolvins, protectins and maresins). There is a temporal shift in the lipid mediator synthesis from the initiation of inflammation to the resolution that allows the formation of different lipid mediators at different times (**Figure 1**). Here we have focused on SPMs, which are of great interest since they permit a return to homeostasis (Serhan et al., 2015; Sugimoto et al., 2016). Charles N. Serhan first identified these SPMs at the periphery and characterized their anti-inflammatory and pro-resolutive properties (Serhan et al., 2000). SPMs actively orchestrate and finely tune the inflammatory response. They decrease pro-inflammatory cytokines and increase anti-inflammatory cytokines, accelerate the phagocytosis of cellular debris and dead cells without immune suppression. A failure in the resolution of inflammation is detrimental for tissue and contributes to a chronic inflammatory response.

## Biosynthesis and Biological Roles of n-3 PUFA-Derived SPMs

### Biosynthesis of n-3 PUFA-Derived SPMs

**Figure 2:**
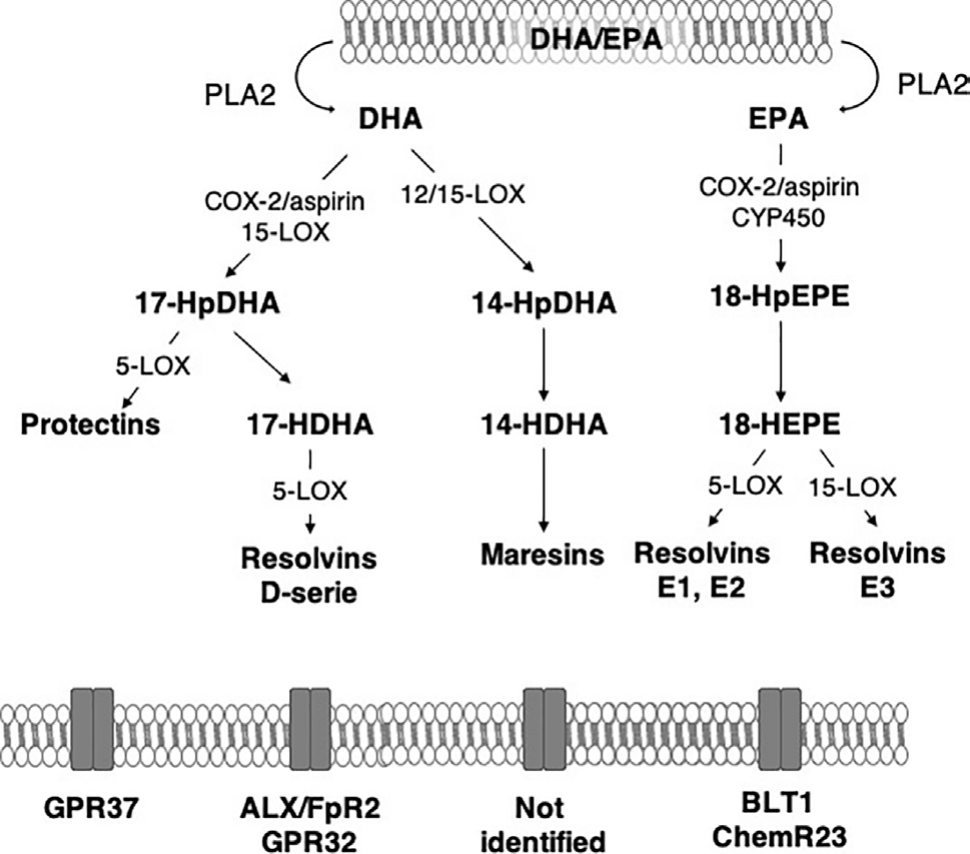
Main synthesis pathway of n-3 long-chain PUFA-derived lipid mediators. ALX/FpR2, lipoxin A4 receptor/formyl peptide receptor 2; BLT1, Leukotriene B4 receptor 1; ChemR23, chemokine-like receptor 1; COX-2, cyclooxygenase-2; CYP450, monoxygenases cytochrome P450; DHA, docosahexaenoic acid; EPA, eicosapentaenoic acid; GPR, G protein-coupled receptor; HDHA, hydroxy-docosahexaenoic acid; HEPE, hydroxy-eicosapentaenoic acid; HpDHA, hydroperoxyl-docosahexaenoic acid; HpEPE, hydroperoxy-eicosapentaenoic acid; LOX, lipoxygenases; PLA2, phospholipase A2.

**Figure 3:**
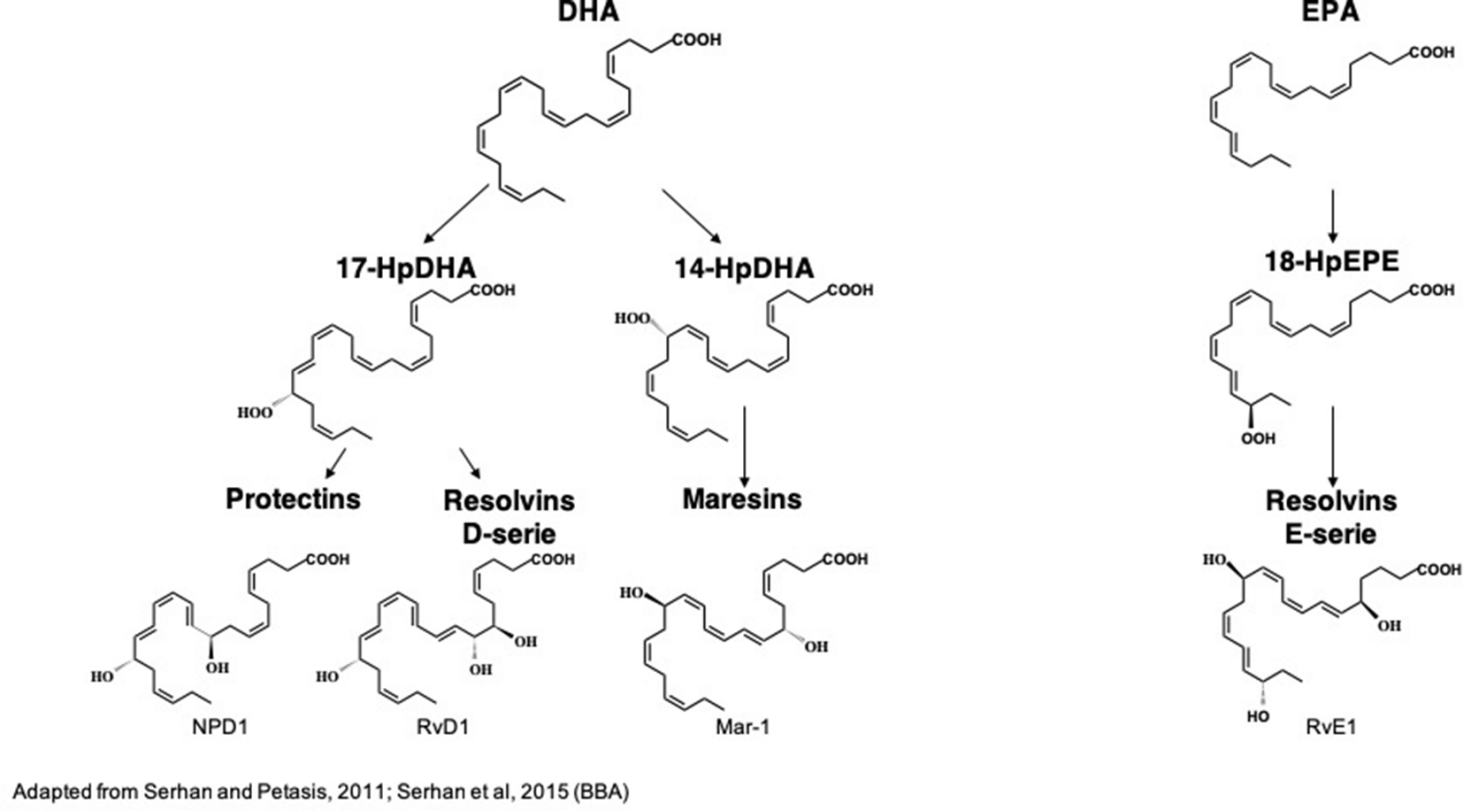
Biochemical structures of the main n-3 long-chain PUFA-derived lipid mediators. DHA, docosahexaenoic acid; EPA, eicosapentaenoic acid; HpDHA, hydroperoxyl-docosahexaenoic acid; HpEPE, hydroperoxy-eicosapentaenoic acid; Mar1, maresin 1; NPD1, neuroproetectin D1; RvD1, resolvin D1; RvE1, resolvin E1. (Serhan and Petasis, 2011; Serhan et al., 2015)

Free (unesterified) n-3 LC-PUFAs are released from membrane phospholipids through the action of phospholipases A2 (PLA2) in response to stimulation. DHA is hydrolyzed by calcium independent PLA2 (iPLA2) from phospholipids and plasmenylethanolamine-PLA2 from plasmalogens (Farooqui and Horrocks, 2006). After this step, n-3 LC-PUFAs undergo an enzymatic conversion to generate SPMs (**Figures 2** and **3**).

n-3 PUFA-derived SPMs are synthesized mainly from DHA and EPA *via* COX-2, lipoxygenases (LOX) and CYP450 monoxygenases (CYP450). In the brain, 15-LOX, 12/15-LOX and 5-LOX are the most abundant LOX and are widely distributed, suggesting the potential production of SPMs (Shalini et al., 2017). They are expressed in the second step of inflammation in the hippocampus (Czapski et al., 2010; Birnie et al., 2013). 15-LOX is involved in neurodegeneration and neurotoxicity due to the increased oxidative stress it generates in models of Alzheimer’s disease (Pratico et al., 2004; Wang et al., 2015) and brain ischemia (Yigitkanli et al., 2017). However, it is also implicated in neuroprotection (Sun et al., 2015). Indeed, it increases the production of 12-HETE and 15-HETE that promote the activation of PPARγ that is neuroprotective through its anti-inflammatory properties. The inhibition of 15-LOX induces hippocampus-dependent cognitive alterations (Shalini et al., 2017). Indeed, it prevented long term potentiation, a major molecular mechanism that describes the strengthening of synapses and underlies learning and memory. Moreover, 15-LOX deletion drastically decreases SPM production in the brain while SPMs also modulate synaptic plasticity (Park et al., 2011; Shalini et al., 2017). In the brain, the inducible COX-2 is activated *via* an NFκB pathway (Nadjar et al., 2005). COX-2 catalyzes the first step of the synthesis of prostaglandins and thromboxanes derived from n-6 PUFAs that contribute to the initiation of inflammation (Davidson et al., 2001; Salinas et al., 2007; Engstrom et al., 2012). COX-2 also produces hydroxyl fatty acids from DHA in both an aspirin-dependent and aspirin-independent manner (Serhan et al., 2002; Oh et al., 2011; Gabbs et al., 2015; Nebert, 2017). In response to LPS, COX-2 is rapidly expressed in the hippocampus (Czapski et al., 2010; Rey et al., 2019). Inhibition of COX-2 delays resolution of acute inflammation (Schwab et al., 2007). CYP450s play a complex role in inflammation by producing n-6 PUFA-derived bioactive mediators such as epoxides that are anti-inflammatory in monocytes and macrophages (Bystrom et al., 2011; Fleming, 2011; Nebert et al., 2013; Gilroy et al., 2016).

DHA is the precursor of resolvins D1-6 (RvD1-6), neuroprotectin D1 (NPD1) and maresins 1–2 (Mar1-2) which all have pro-resolutive and anti-inflammatory properties (Spite and Serhan, 2010; Halade et al., 2018). RvD1-6 are synthesized from DHA but RvD1 is the most studied because it has powerful anti-inflammatory and pro-resolutive properties. DHA is converted into monohydroxy DHA, 17-hydroxy docosahexaenoic acid (17-HDHA) by acetylated COX-2, CYP450 and 15-LOX (Barden et al., 2016; Halade et al., 2018) and then into RvD1 by 5-LOX (Sun et al., 2007; Recchiuti, 2013). DHA is also converted into di-hydroxy-DHA, termed protectin D1 (PD1) or neuroprotectin D1 (NPD1) when produced in the central nervous system by 5- and 15-LOX (Hong et al., 2003; Aursnes et al., 2014; Kuda, 2017; Doyle et al., 2018). Acetylated COX-2 permits the synthesis of aspirin-triggered PD1 (AT-PD1) which has powerful protective effects (Bazan et al., 2012). DHA is transformed into Mar 1-2 by 12/15-LOX *via* the synthesis of 14-HDHA (Serhan et al., 2009; Barden et al., 2016; Halade et al., 2018).

EPA is the precursor of resolvins E1 (RvE1), E2 and E3 that have many biological roles (Serhan et al., 2000; Rey et al., 2016; Halade et al., 2018). It is converted by aspirin-triggered acetylated COX-2 or CYP450 into 18R-hydroxyeicosapentaenoic acid (18R-HEPE), that is transformed into RvE1 or E2 by 5-LOX (Ohira et al., 2010; Barden et al., 2016) or into RvE3 by 15-LOX (Isobe et al., 2012).

Little is known concerning the pharmacokinetics and dynamics of oxylipins. They are synthesized *in situ*, reinforcing the importance of the fatty acid composition of the brain. They are not stored, but produced on demand and they are unstable, being rapidly metabolized.

The structure of all derivatives is highly preserved in the evolution from fish to humans suggesting their great bioactive role in all organ systems. Dysfunction of SPM production can be due to insufficient EPA and DHA supply leading to inadequate production of SPMs or to the polymorphism of the enzymes involved in their synthesis or to a defect in the binding of SPMs to their receptors (Sugimoto et al., 2016). In humans, reduced SPM production is associated with chronicity and the magnitude of persistent inflammation. SPMs have multiple biological roles in the maintenance of homeostasis.

### Biological Roles of DHA-Derived SPMs

In human serum, the DHA-derivatives represent 30.7% of the identified SPMs (Colas et al., 2014; Serhan et al., 2018). RvD1, PD1 and Mar1 were detected at 30.9 pg/mL, 5.6 pg/mL and 21.2 pg/mL, respectively. They act in the nanomolar or picomolar range as compared with DHA and EPA acting in the micromolar range (López-Vicario et al., 2016; Clària et al., 2017; Serhan, 2017a; Wang and Colgan, 2017; Rosenthal et al., 2018).

#### Resolvins

RvDs have been identified in mice peritoneal exudates (Serhan et al., 2002). They have many properties at the periphery: protection against bacterial infection, prevention of leucocyte infiltration, inhibition of the production of cytokines, etc. (Spite et al., 2009; Serhan and Petasis, 2011; Chiang et al., 2012; Dalli et al., 2013a; Winkler et al., 2016). RvD1 and its precursor metabolites have been detected in the brain.

*In vitro*, 17-HDHA has been found in glial cells after DHA and inflammatory stimulus (Hong et al., 2003). *In vivo*, RvD1 has been identified in mouse brain following cerebral ischemia (Marcheselli et al., 2003). Intravenous (iv) DHA injection increases RvD1 levels in rats suggesting the conversion of DHA into RvD1 (Mulik et al., 2016). RvD1 acts through the lipoxin A4 receptor/formyl peptide receptor 2 (ALX/Fpr2) in rodents and the G protein coupling receptor 32 (GPR32) in humans (Recchiuti, 2013) in the picomolar range but induces biological effects in the nanomolar range (Sun et al., 2007; Krishnamoorthy et al., 2010). An overexpression of ALX/Fpr2 or GPR32 increases in phagocytosis by macrophages whereas their deletion decreases the phagocytosis response (Perretti et al., 2002; Krishnamoorthy et al., 2010; Cooray et al., 2013). RvD1 regulates the expression of specific micro-RNA (miR) to control the intensity and the length of inflammation *via* the regulation of target genes such as inflammatory cytokine genes (Fredman and Serhan, 2011; Recchiuti et al., 2011; Rey et al., 2016; Bisicchia et al., 2018). RvD1 increases the expression of miR-21, miR-146b and miR-219 and decreases the expression of miR-208a in macrophages and peritoneal exudates in mice (Recchiuti et al., 2011; Krishnamoorthy et al., 2012). RvD1 modulates miR-155, miR-146, miR-21 and miR-219 in microglial cells (Rey et al., 2016). These miRs have different biological roles: miR-21 is essential to the production of anti-inflammatory cytokine IL-10, miR-146 regulates the transcription of cytokines, chemokines and their receptor, miR-219 decreases the transcription of TNF-α and miR-208a regulates the activation of NFkB (Recchiuti et al., 2011).

RvD1 controls the inflammatory response in many animal models *via* its anti-inflammatory and pro-resolutive properties. In rats, endogenous RvD1 levels decrease at the beginning of inflammation and then increase during the resolution phase (Sun et al., 2014). Recent studies describe the anti-inflammatory properties of RvD1 in microglia and astrocyte cell cultures (Abdelmoaty et al., 2013; Li et al., 2014; Rey et al., 2016). Indeed, in BV2 microglia cell culture, RvD1 promotes the IL-4-induced M2 phenotype (Li et al., 2014) and inhibits LPS-induced pro-inflammatory cytokines (Rey et al., 2016). In astrocyte cell culture, RvD1 attenuates LPS-induced TNF-α (Abdelmoaty et al., 2013). *In vivo*, in a model of remote damage, Bisicchia et al. recently show in rats that RvD1 promotes functional recovery and reduces neuroinflammation *via* miRs (Bisicchia et al., 2018).

##### Biological Roles of RvD1 in Humans

The effect of RvD1 has been studied in patients suffering from Alzheimer’s disease. This pathology is characterized by an increase in microglial activation and in pro-inflammatory cytokine production in the brain (Griffin et al., 1989; Cagnin et al., 2001). Interestingly, RvD1 levels in the cerebrospinal fluid are positively correlated with the enhancement of cognitive functions of patients with dementia (Wang et al., 2015). Indeed, RvD1 may be involved in Aβ phagocytosis. This has been shown *in vitro* in macrophages isolated from Alzheimer’s patients (Mizwicki et al., 2013; Famenini et al., 2017). Thus the decrease in RvD1 levels in Alzheimer patients’ brains could contribute to the evolution of the disease.

##### Biological Roles of RvD1 in Rodents

RvD1 attenuates the pro-inflammatory status in the central nervous system. Indeed, an intrathecal injection of 17R-HDHA decreases TNF-α release in the spinal cord in rats (Abdelmoaty et al., 2013). Orr et al. show that icv injection of 17S-HDHA into mice decreases the expression of hippocampal pro-inflammatory cytokines IL-1β and TNF-α induced by LPS acute icv injection (Orr et al., 2013). However, these authors detected NPD1 but did not detect RvD1, suggesting that the effect of 17S-HDHA was rather due to the conversion of 17S-HDHA into NPD1. RvD1 is also able to stimulate phagocytosis in macrophages (Rossi et al., 2015). Indeed, DHA and RvD1 induce the polarization of macrophages toward an M2 phagocytic phenotype in mice (Titos et al., 2011).

Studies have highlighted the protective role of RvD1 in the occurrence of cognitive deficits. Terrando et al. showed that an ip injection of 17-HDHA restores transmission and synaptic plasticity and prevents astrogliosis and cognitive decline in a systemic inflammation model in mice (Terrando et al., 2013). Conversely, an inhibition of 15-LOX, associated with a decrease in RvD1, alters synaptic plasticity and working memory as demonstrated in rats (Shalini et al., 2017). RvD1 plays also a role during the recovery phase following cerebral ischemia. Indeed, the precursor of 17-HDHA, 17-hydroperoxy docosahexaenoic acid (17-HpDHA), accumulates in the hippocampus of mice (Marcheselli et al., 2003). An ip chronic administration of 17R-HDHA synthesized by acetylated COX-2, prevents cognitive deficits and attenuates motor disorders but doesn’t ameliorate microglial activation and sleep quality in mice (Harrison et al., 2015). Moreover, Fat-1 mice that have more brain n-3 LC-PUFAs, have higher hippocampus RvD1 levels, associated with less cognitive deficits, a better neuronal survival, a decrease in astrocyte and microglial activation and a reduction in pro-inflammatory status following brain ischemia (Luo et al., 2014; Delpech et al., 2015a).

Studies have also highlighted the protective role of resolvins in the depressive-like behavior in rodents. Some of them have been recently reported by Furuyashiki et al. (Furuyashiki et al., 2019). An icv injection of RvD1, D2, E1, E2, or E3 significantly decreases LPS-induced depressive-like behavior in mice (Deyama et al., 2017; Deyama et al., 2018a; Deyama et al., 2018b). Moreover, the occurrence of depressive-like behavior associated with pain can also be prevented by an intrathecal injection of 17R-HDHA that is associated with the decrease of pain perception and a restoration of dopamine and glutamate levels in the brain both in rats (Abdelmoaty et al., 2013) and mice (Klein et al., 2014). RvD1 and D2 have also positive effects in chronic mild stress-induced depression and in post-myocardial infarct depression in rats (Gilbert et al., 2014) and mice (Ishikawa et al., 2017).

##### Biological Roles of RvD1 in In Vitro Models

The effects of RvD1 were tested on different brain cells. In microglial cells, RvD1 potentiates the effect of the anti-inflammatory cytokine IL-4 in the activation of M2 phenotype of microglia (Li et al., 2014). Moreover, we showed that RvD1 decreases LPS-induced pro-inflammatory cytokines (TNF-α, IL-6 and IL-1β) gene expression in microglial BV2 cells *via* the modulation of miRs (Rey et al., 2016). This suggests its pro-resolutive activity in microglia. In astrocytes, RvD1 decreases TNF-α release induced by LPS injection (Abdelmoaty et al., 2013). In neurons from spinal nods, RvD1 increases neurite outgrowth (Shevalye et al., 2015). All these studies suggest that RvD1 can play a central role in the regulation of neuroinflammatory pathologies.

Other D resolvins have been identified in rodent brain: RvD2, RvD4 and RvD5 (Orr et al., 2013; Hashimoto et al., 2015; Winkler et al., 2016). RvD2 limits the activation of microglial cells and inhibits the TLR4/NFκB pathway (Tian et al., 2015). Moreover, chronic intrathecal RvD2 injection prevents the behavioral alterations induced by LPS central injection (Tian et al., 2015). It also prevents depressive-like behavior induced by ip LPS injection by regulating mammalian target of rapamycin complex 1 (mTORC1) complex (Deyama et al., 2017). RvD5 level is decreased in the brain of Alzheimer’s disease patients but its role has not yet been identified (Zhu et al., 2016).

#### Neuroprotectin

Di-hydroxy-DHA termed protection D1 (PD1) has been identified in blood, peritoneal neutrophils and neuroprotectin D1 (NPD1) in the brain in response to zymosan in mice (Hong et al., 2003). Marcheselli et al. have measured NPD1 production in mice hippocampus following brain ischemia (Marcheselli et al., 2003). One receptor for NPD1 has recently been identified as GPR37 (Qu and Caterina, 2018). NPD1 inhibits the oxidative stress in retinal epithelial cells and stimulates their proliferation (Bazan, 2009; Calandria and Bazan, 2010). NPD1 is also able to inhibit neovascularization *via* microglial cell ramification in mice (Sheets et al., 2013). In the central nervous system of mice, the NPD1 level greatly increases in the hippocampus following brain ischemia or acute central LPS injection (Marcheselli et al., 2003; Orr et al., 2013). Hence, NPD1 limits neutrophil infiltration, inhibits NFκB and then decreases pro-inflammatory gene expression (Marcheselli et al., 2003; Bazan et al., 2012; Yao et al., 2013). NPD1 levels decrease in the hippocampus of Alzheimer’s disease patients (Lukiw et al., 2005). It plays a role in cellular survival *via* anti-apoptotic protein induction and attenuates pro-inflammatory responses following Aβ exposure *via* NFκB regulation (Lukiw et al., 2005; Bazan, 2008; Asatryan and Bazan, 2017).

#### Maresins

Mar1 has been identified in mice peritoneal macrophages (Dalli et al., 2013b; Serhan et al., 2009). Its receptor has not been identified yet (Zhu et al., 2016). Mar1 is involved in the resolution of inflammation, prevents neutrophil infiltration, and increases the phagocytosis of apoptotic neutrophils by macrophages in a peritonitis murine model (Serhan et al., 2009). In a murine model of colitis, Mar1 decreases the expression of pro-inflammatory cytokines by inhibiting the NFκB pathway and activating the M2 phenotype in macrophages (Marcon et al., 2013). Mar1 and its precursor 14-HDHA have recently been identified in the hippocampus of mice (Orr et al., 2013). In *post-mortem* brain of Alzheimer’s disease patients, the Mar1 level is decreased (Zhu et al., 2016). In this pathology, its role is to stimulate Aβ plaque phagocytosis by microglial cells and to decrease inflammatory marker levels. Hence, Mar1 may play an important role in the pathogenesis of Alzheimer’s disease (Zhu et al., 2016). In cerebral ischemia in mice, Mar1 icv injection decreases inflammation and mitochondrial damage and also reduces neurological deficits *via* activation of SIRT-1 signaling (Xian et al., 2016; Xian et al., 2019). After spinal cord injury in mice, iv injection of Mar1 promotes resolution of inflammation (reducing pro-inflammatory cytokines, silencing pro-inflammatory signaling cascades and enhancing the M2 repair macrophage phenotype) and functional recovery (Francos-Quijorna et al., 2017). Mar1 also decreases *in vitro* neuronal death (Zhu et al., 2016).

### Biological Roles of EPA-Derived SPMs

RvE1, and its precursor 18-HEPE, have been detected in the hippocampus of rats (Chen et al., 2011) and mice (Orr et al., 2013; Siegert et al., 2017). In human serum, the EPA-derivatives represent 25.9% of the identified SPMs (Colas et al., 2014; Serhan et al., 2018).

RvE1 has been initially identified in mouse exudates (Serhan et al., 2000). RvE1 directly binds to its receptor ChemR23 or CMKLR1 (chemokine-like receptor 1) (Ohira et al., 2010). It is also a partial agonist of LTB4 receptor (BLT1) (Arita et al., 2007). In the central nervous system, ChemR23 has been identified in the prefrontal cortex, hippocampus and brainstem (Guo et al., 2012), in microglial cells (Graham et al., 2009; Rey et al., 2016) and in neurons (Xu et al., 2010). It is highly expressed in neurons, in microglial cells and in astrocytes in the *post-mortem* hippocampus of Alzheimer’s patient brain (Wang et al., 2015). This increase could be due to a compensatory mechanism to counter-balance the decrease in RvE1 in such patients (Wang et al., 2015). *In vitro*, RvE1 plays also a direct role in microglial cells by inhibiting microglial activation and pro-inflammatory cytokine release (Xu et al., 2013; Rey et al., 2016). RvE1 and its precursor, 18R-HEPE, exert anti-inflammatory and anti-apoptotic properties (Hecker et al., 2018). Indeed, they restore mitochondrial dysfunction induced by inflammation in mononuclear blood cells. *In vivo*, RvE1 modulates the inflammatory profile and microglial activation in mice (Xu et al., 2013; Harrison et al., 2015). Intraperitoneal injection of RvE1 also modulates inflammation (by reducing Il-1β, Il-6 and IL-10 levels in the prefrontal cortex) and decreases the measures of Aβ pathology in a murine model of Alzheimer’s disease (Kantarci et al., 2018). RvE1 and RvE2 centrally administered also reduce the LPS-induced depressive-like behavior through ChemR23 in mice (Deyama et al., 2018b). RvE1, a total agonist of ChemR23, is more effective than RvE2, which is only a partial agonist of this receptor (Serhan and Chiang, 2013).

## Regulation of the n-3 PUFA-Derived SPMs

Resolution of inflammation is an active process involving the regulation of the synthesis of numerous mediators in a tightly coordinated manner. The balance between n-3 PUFA-derived SPMs and the pro-inflammatory mediators determines the duration of the inflammatory response and the timing of resolution (Fredman et al., 2017).

### Regulation of the n-3 PUFA-Derived SPMs by PUFA Consumption

We have previously shown that PUFA consumption leads to modifications in PUFA levels in the brain. The PUFA derivative levels and their biosynthetic enzyme expression also depend on dietary PUFAs.

**Figure 4:**
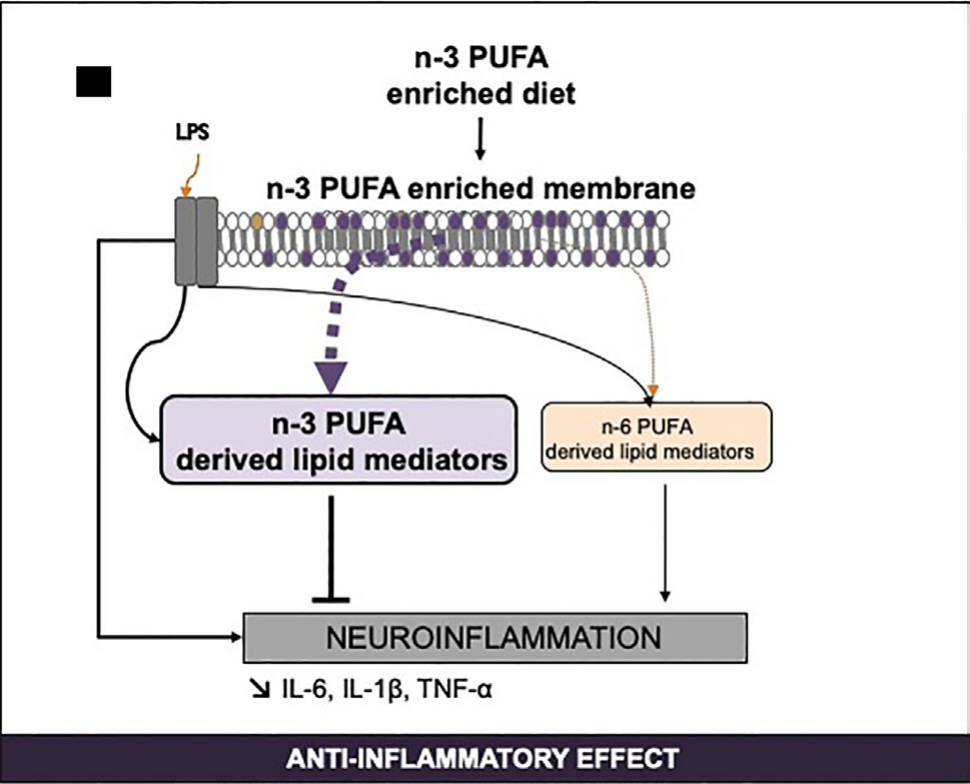
Regulation of n-3 long-chain PUFA-derived SPMs by PUFA consumption and inflammation. IL, interleukine; LPS, lipopolysaccharides; PUFA, polyunsaturated fatty acids; SPM, specialized pro-resolving mediators; TNF, tumor necrosis factor.

SPM levels in peripheral organs and the brain are modulated by different dietary supplies of PUFAs (**Figure 4**). In humans supplemented with 2.4g/day of n-3 LC-PUFAs for 3 weeks (EPA+DHA), SPM levels are generally found to be between 20 and 200 pg/mL (Mas et al., 2012). A shorter supplementation period (5 days) also increased DHA- and EPA- derived SPMs in plasma, especially 18-HEPE, RvE1, 17- and 14-HDHA (Barden et al., 2014). In another study, supplementing patients with a higher fish oil supplement (17.6g/day EPA+DHA) for 24h, only PD1 is detected in the plasma and this increases from 1.0–1.2 pg/mL to 2.7–4.1 pg/mL (Skarke et al., 2015; Barden et al., 2016). These differences in SPM levels in human plasma could be due to differences in study design, dose, duration of n-3 fatty acid supplementation and patient characteristics, etc. (Barden et al., 2016; Chiang and Serhan, 2017). Moreover, dietary supplementation in DHA (1.7g/j) and EPA (0.6g/j) for 6 months increased the RvD1 released by blood mononuclear cells in Alzheimer’s disease patients as compared with controls (Wang et al., 2015). Results from intervention studies show that EPA and DHA dietary supplementation increases EPA- and DHA-derived oxylipins although with high inter-individual variances (Ostermann et al., 2017).

In animals, a 3-week fish oil supplementation in arthritic mice increases synthesis of n-3 PUFA-derived SPMs associated with a diminished production of pro-inflammatory mediators (Norling et al., 2016). n-3 PUFA dietary supplementation (140 mg EPA+110 mg DHA/kg animal) in aged rats for 10 weeks enhances the production of lipid mediators derived from DHA and EPA in the cerebral cortex (Hashimoto et al., 2015). DHA and EPA supply also decreases the n-6 PUFA-derived metabolites (which are mainly pro-inflammatory) and increases the n-3 PUFA-derived metabolites in the plasma and at the periphery both in mice (Balvers et al., 2012; Lazic et al., 2014) and rats (Hashimoto et al., 2015). A diet enriched with fish oil (containing 9% EPA + 9% DHA) for 8 weeks in mice increases plasmatic RvD1 (Shevalye et al., 2015). In Fat-1 mice, brain EPA increase is accompanied by an increase in EPA-derived HEPEs (Siegert et al., 2017). Conversely, an n-6 PUFA dietary supply from weaning to 15 weeks of age increases the n-6 PUFA metabolites and decreases the EPA-derived metabolites in the cortex and the plasma of rats (Taha et al., 2016). Recently, we showed that, in mice, n-3 LC-PUFA supplementation from weaning for 2 months induces n-3 LC-PUFA enrichment in the hippocampus and subsequently an increase in n-3 PUFA-derived SPMs and a decrease in n-6 PUFA-derived mediators (Rey et al., 2019). Moreover, in response to LPS, n-3 LC-PUFA-deficient mice present a pro-inflammatory lipid mediator profile whereas n-3 LC-PUFA-supplemented mice display an anti-inflammatory profile in the hippocampus (Rey et al., 2019). Indeed, the consumption of n-3 LC-PUFAs increases the EPA and DHA derivatives. Hashimoto et al. found the same in the cortex of aged rats (Hashimoto et al., 2015). These results differ from those of Trépanier et al. who found that, in a model of Fat-1 mice, a greater increase in brain DHA induced by dietary supply has no effect on the resolution of inflammation after an icv LPS injection (Trépanier et al., 2018).

SPM production is also finely tuned by the regulation of the enzymes in their biosynthesis pathway. A 15-week n-3 PUFA-deficient diet increases the COX-2 expression in the prefrontal cortex of rats, suggesting an increase in AA-derived pro-inflammatory mediator levels (Rao et al., 2007). Conversely, a 15-week n-6 PUFA-deficient diet decreases brain COX-2 expression in rats (Kim et al., 2011). Interestingly, Taha et al. showed that rats fed on an n-6 PUFA-deficient diet for 15 weeks are protected from an increase in COX-2 observed in LPS-treated n-3 PUFA-deficient animals (Taha et al., 2017). However, we showed that 2 months’ n-3 LC-PUFA dietary supplementation was not sufficient to modulate COX-2 expression in the brain of mice (Rey et al., 2019). An n-3 LC-PUFA diet increases 15-LOX expression in the hippocampus without changes in the 5-LOX expression (Gabbs et al., 2015). The increase in 15-LOX in the brain has also been described following a 15-week n-6 PUFA-deficient diet in rats (Kim et al., 2011). 15-LOX is responsible for many n-6 and n-3 PUFA derivatives. Those synthesized from AA drastically decrease, and those produced from DHA increase, following a diet enriched in n-3 PUFAs in both mice and rats (Ostermann et al., 2017; Shalini et al., 2017; Rey et al., 2019).

Results on 15-LOX functions on inflammation regulation are conflicting. 15-LOX was initially described as deleterious in neurodegenerative pathologies because it increased the oxidative stress and neuronal degeneration (Pratico et al., 2004; Chinnici et al., 2007; Di Meco et al., 2014; Wang et al., 2015; Yigitkanli et al., 2017). It alters the mitochondrial function of neurons and then induces neuronal death (Pallast et al., 2009). However, recent studies demonstrate the protective role of 15-LOX, particularly in cognition. An increase in its expression is associated with a better working memory (Sun et al., 2015; Shalini et al., 2017). Moreover, an increase in 15-LOX expression during cerebral ischemia is essential for the recovery of neurological functions after the ischemic event (Wang et al., 2017). These results suggest that 15-LOX has a beneficial role in acute inflammation (ischemia, LPS) and is deleterious in chronic inflammation (neurodegenerative pathologies). SPM production is also partly regulated by the availability of free PUFAs that are their precursors. Indeed, PLA2 activity also depends on PUFA dietary supply. An n-6 PUFA-deficient diet increases the iPLA2 that is responsible for the release of DHA from the membranes and a decrease in calcium sensitive cytosolic PLA2 (cPLA2) associated with the hydrolysis of AA from the membranes (Kim et al., 2011).

Lipid nutrition, to which people are exposed throughout their lives, seems to play a major role in the synthesis of bioactive SPMs.

### Regulation of the n-3 PUFA-Derived SPMs by Inflammation

Numerous studies have highlighted that inflammation modulates lipid mediator synthesis at the periphery and in the brain (**Figure 4**).

In humans, Wang et al. showed that the RvD1 level in the cerebrospinal fluid (CSF) of Alzheimer’s disease patients is positively correlated with cognitive function (Wang et al., 2015). Moreover, 15-LOX was decreased in the CSF suggesting an alteration of the resolution of inflammation in Alzheimer’s patients.

In rats, brain ischemia increases the production of 5 of mono-, di- and tri-hydroxy-DHA derivatives (Farias et al., 2008; Hennebelle et al., 2017). However, LPS icv injection does not impact the RvD1 level (Rosenberger et al., 2004; Taha et al., 2017). We have recently shown that, in mice, ip LPS injection modifies the n-6 PUFA derivative profile but not n-3 LC-PUFA derivatives (Rey et al., 2019).

*In vitro*, most studies have been performed on peripheral immune cells. The lipid mediator profile in rodent neutrophils and macrophages changes with macrophage phenotype M1 or M2, or with the inflammatory stimulus in neutrophils (Dieter et al., 2002). Indeed, M2 macrophages produce more D resolvins, protectins and maresins and less AA-derived pro-inflammatory mediators than M1 macrophages (Dalli and Serhan, 2012).

Inflammation more drastically alters the expression of biosynthetic enzymes than an n-3 LC-PUFA dietary supply. In rats, traumatic brain injury increases the COX-2, 5-LOX, 15-LOX and CYP450 expression in the hippocampus and cortex, suggesting an alteration of all lipid mediator biosynthesis pathways (Birnie et al., 2013). LPS also regulates the expression of these enzymes. An icv LPS injection increases COX-2 expression and activity in the brain in mice (Rey et al., 2019) and rats. Moreover, in *in vitro* human monocytes and dendritic cells, anti-inflammatory cytokines IL-4 or IL-13 increase the production of 15-LOX and decrease the production of 5-LOX (Nassar et al., 1994; Spanbroek et al., 2001).

Neuroinflammation also modifies SPM receptor expression. Indeed, we show that LPS increases significantly the expression of RvD1 and RvE1 receptors (ALX/Fpr2 and ChemR23, respectively) in BV-2 microglial cells (Rey et al., 2016). The increase in ALX/Fpr2 expression was also detected in monocytes, in the hippocampus and in the cortex and in microglial cells in response to an inflammatory stimulus (Krishnamoorthy et al., 2010; Wang et al., 2011; Wang et al., 2015; Guo et al., 2016). Then, inflammation activates the SPM signaling pathway in the brain to regulate the inflammatory response.

## Human Translation

Several possibilities can be considered to translate the findings described above and then attenuate the inflammatory tone, amplitude and duration of inflammation. The first one is to increase the local production of n-3 LC-PUFA-derived SPMs. We see that dietary means is a good way to modulate the level of the fatty acids from which they are synthesized and then to modify their synthesis. The SPM profile synthesized by each individual could be responsible for the differences in the effects of n-3 PUFA obtained in humans. SPM profiles should be established in patients with different acute and chronic inflammatory pathologies, and in mice under the same conditions to find markers of neuroinflammation in the plasma that can be transposed to humans. Thanks to new technologies in liquid chromatography mass spectrometry (LC-MS/MS), specific mediators produced during physiological and non-physiological conditions should be identified, allowing patient stratification according to disease severity. It could be interesting to determine an individual metabolomic profile to define personalized nutrition (n-3 PUFAs and doses) allowing an increase of n-3 PUFA-derived SPMs in the target tissue. Indeed, there are individual differences in diets and in n-3 PUFA supplementation and also in nutrient metabolism and biological responses to food/nutrients. The aim of personalized nutrition is to increase health using nutrition by delivering specific personalized intervention suited to each individual based on the individual’s nutritional phenotype, metabolic profile, and environmental factors in order to prevent and treat chronic disease. Personalized nutrition can also be applied to healthy people. It is nowadays accessible because of a better understanding of the mechanisms of the effect of nutrition on health and also because of the progress in technologies enabling the identification of specific markers. Personalized nutrition has already shown its efficacy, especially in the Food4Me study involving >1600 participants from 7 European countries and in a systematic review and meta-analysis showing a greater efficacy of personalized nutrition in changing diet than a conventional approach (Celis-Morales et al., 2018).

The second possibility for taking advantage of research on the resolution of inflammation is to administer exogenous SPMs. Serhan defines a new concept of resolutive pharmacology targeting the development of SPM analogs, resistant to local inactivation, to stimulate natural circuits of resolution (Chiang and Serhan, 2017). The objective of this new therapeutic pathway is to administer these analogs in association with classical therapy in order to decrease the doses, thus limiting the secondary effects. A clinical trial has reported for the first time the efficacy of an RvE1 analog in patients with dry eye symptoms (Resolvyx Pharmaceuticals). These encouraging results should be extended to the use of such molecules to treat other inflammatory diseases.

## Conclusion

In the investigation of new anti-inflammatory treatments without the secondary effects of traditional therapy, SPMs are promising therapeutic compounds: they are of natural origin and are active at low concentrations (nM) as compared with their precursor (µM) (Ariel and Serhan, 2007; Bannenberg and Serhan, 2010). SPMs are detectable in the brain and their level can be modulated by dietary supplementation. They have potent anti-inflammatory and pro-resolutive properties and we confirm the main role of nutrition as an environmental factor that greatly influences the inflammatory response. It is important to determine if inflammatory pathologies are due to unresolved inflammation, attributed to a decrease in n-3 LC-PUFA dietary intake leading to a decrease in SPM levels, to an enzyme or receptor polymorphism, to a de-regulation of SPM receptors or to a decrease in their expression. We should also determine if these mechanisms could be restored by n-3 LC-PUFA or SPM analog administration.

## Author Contributions

All authors (CJ, CR, SL) contributed to the writing of the manuscript.

## Conflict of Interest Statement

The authors declare that the research was conducted in the absence of any commercial or financial relationships that could be construed as a potential conflict of interest.
